# Chagas disease research and development: Is there light at the end of the tunnel?

**DOI:** 10.1016/j.csbj.2016.12.002

**Published:** 2016-12-14

**Authors:** Eric Chatelain

**Affiliations:** Drugs for Neglected Diseases *initiative* (DND*i*), 15 Chemin Louis Dunant, 1202 Geneva, Switzerland

**Keywords:** *Trypanosoma cruzi*, Drug discovery and development, Challenges

## Abstract

Chagas disease, or American trypanosomiasis, is the result of infection by the parasite *Trypanosoma cruzi*. It is endemic in Latin America, and spreading around the globe due to human migration. Although it was first identified more than a century ago, only two old drugs are available for treatment and a lot of questions related to the disease progression, its pathologies, and not to mention the assessment of treatment efficacy, are subject to debate and remain to be answered. Indeed, the current status of evidence and data available does not allow any absolute statement related to treatment needs and outcome for Chagas patients to be made. Although there has been some new impetus in Research and Development for Chagas disease following recent new clinical trials, there is a scientific requirement to review and challenge the current status of evidence and define basic and clinical research priorities and next steps in the field. This should ensure that the best drugs for Chagas disease are developed, but will require a focused and collaborative effort of the entire Chagas disease research community.

## 1 Introduction

Chagas disease (CD), also known as American trypanosomiasis [1], [2] is an important public health problem not only in Latin America where it is endemic in 21 countries but it is increasingly spreading in other areas such as Europe, North America, Japan and Australia, mainly due to migration [3], [4]. Around 6 million people are affected worldwide and approximately 7000 deaths occur annually, making CD the major cause of death from a parasitic disease in Latin America and a significant contributor to the global burden of cardiovascular disease, with CD the main cause of infectious cardiomyopathy in the world [5], [6], [7]. Health issues associated with CD, such as reduced worker productivity and mortality, have a significant economic burden amounting to a loss of more than half a million Disability-Adjusted Life Years and an annual cost of several billions U.S. dollars, depending on the estimate [8], [9]. CD affects mostly people living in poverty in remote areas and as such less than 1% of infected people have access to diagnosis and treatment.

The disease develops following infection by the protozoan parasite *Trypanosoma cruzi* (*T*. *cruzi*). It is possible to distinguish the following modes of transmission [10], [11], [12], [13]:

– Vectorial: The parasite is transmitted by the Triatomine vector also known as the “kissing bug”
– Congenital: Transmission from mother to child
– Oral: Through the ingestion of contaminated food or drink
– Iatrogenic: Following contaminated blood transfusion or organ transplantation.

**Fig. 1:**
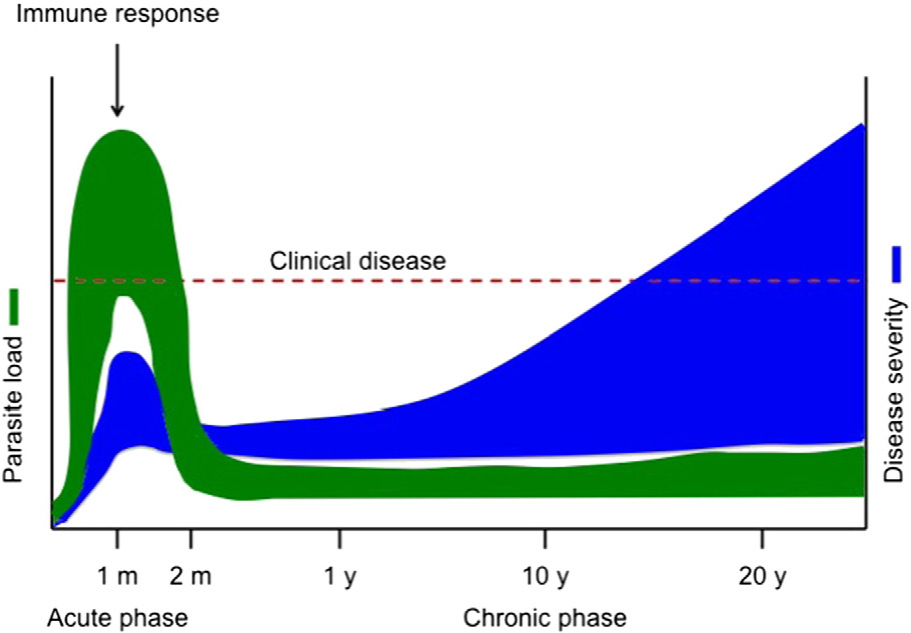
Chagas disease progression (adapted from Tarleton^14^). *Trypanosoma cruzi* infection consists of an acute disease phase characterized by elevated parasite load (green). Immune response brings parasite load down to low/undetectable levels. Chagas disease then progresses to the chronic phase, the severity of which (blue) depends on time since infection and host immune status or genetic background. Thirty to 40% of Chagas patients in the chronic phase will develop clinical manifestations such as cardiomyopathy or megacolon; the remaining 60 to 70% will stay asymptomatic (indeterminate form of the disease). m, month; y, year.

The disease has effectively two clinical phases (see Fig. 1) [14]. The acute phase (fatal for 2–8% of infected people), lasts up to 2 months and is usually asymptomatic or unrecognized. The parasite load peaks at this stage and can be detected easily in blood by direct examination using microscopy or PCR. *T*. *cruzi* proliferates actively in the infected individual and invades many host cell types. The host immune system is then activated, resulting in a dramatic reduction in parasite load with subsequent control of the infection (very low parasite load undetectable by microscopic methods). People then enter the so-called chronic phase of the disease which can be divided into two stages:

– The chronic asymptomatic (or indeterminate) stage which may last for decades after infection, during which patients can transmit the parasite to others. The patient has evidence of immunity (antibodies to specific antigens of *T*. *cruzi*) but remains infected. At this stage infection is controlled, but the immune system does not prevent disease progression.
– The chronic, symptomatic stage that develops in 10 to 40% of infected patients, typically after decades, causes cardiomyopathies and digestive tract pathologies.

CD can also be reactivated if patients in the chronic phase are immune compromised as in the case of co-infection with HIV [15]. Progressive heart failure (70%) and sudden death (30%) remain the main causes of death in these patients [16].

Current treatment for CD, two old nitro-heterocyclic drugs — benznidazole (Abarax/ELEA) and nifurtimox (LAMPIT/Bayer) — are effective against the acute phase of infection. While there is increasing evidence of their efficacy in the chronic indeterminate stage of the disease, the use of these drugs is limited due to their poor availability and side-effects such as allergic dermatitis, pruritus and gastrointestinal manifestations among others [17], [18], [19]. These facts highlight an urgent need for improved access to currently available treatments in the short term but also a clear need for efficacious and safer drugs for the future.

## 2 Still many unanswered questions

Although the illness was first described by Carlos Chagas more than a century ago [20], our understanding of the disease, its pathologies and the factors related to its progression is still limited and a substantial number of fundamental questions remain unanswered [21]. The question “What is Chagas disease?” might be viewed as naïve but considering the complexity of the life-cycle of *T*. *cruzi* parasites, the host/parasite interactions and the disease evolution, it remains a valid question.

Over the years, different theories related to CD progression have emerged, principally the autoimmunity and infectious or parasite persistence theories [22], [23]. However, neither are mutually exclusive and there are data supporting both theories. Most probably, Chagas disease is the result of a mixture of complex interactions between the parasite *T*. *cruzi*, the host immune system and other genetic factors, the details of which are yet to be fully established [24]. Recent experimental data generated using a BioLuminescence Imaging (BLI) murine model of Chagas have shown that infection of the heart by *T*. *cruzi* was transient and occurred in less than 50% of the infected mice. Consideration of the parasite dynamics during infection and the potential non-essentiality of having parasites residing in the heart at all times to induce inflammation of the organ could be a first step to re-unite both theories [25]. However, this is still a subject open to debate and remains to be clearly demonstrated. Indeed, as the authors rightly stated, one cannot exclude that parasites were still present continuously in the cardiac tissue but in numbers too low to be measured by bioluminescence.

It is striking to observe that only a certain percentage (10 to 40% depending on the sources) of those infected in the chronic phase will go on to develop the disease [26], [27], [28]. For a number of reasons, it is extremely difficult to obtain accurate data on this topic both due to the natural history of infection and the challenge of clinical studies specifically addressing this issue. Why then do some develop the disease when most infected people do not? Can we be confident with the data on this topic? Is it just a question of time or duration of infection and accumulation of damage which determines clinical outcome? What determines whether the disease will evolve to cardiomyopathy, megacolon or both?

A recurrent question relates to the duration of treatment with the current available drugs as the rationale for the currently used 60-day or 90-day regimen is very hard to trace. New clinical and experimental data support reducing the dose and duration of treatment with benznidazole but further research on this approach is greatly needed. Altcheh et al. [29] have shown that children treated with benznidazole have a lower exposure to the drug and can be cured. There are some isolated cases and non-controlled studies showing that this could also be the case in adults [30], [31], [32], [33], [34], [35]. New data from a BLI animal model showed recently that chronically infected mice could be cured with only 5 days' treatment with a standard dose of benznidazole and that a reduction of the dose is possible but requires longer treatment [36]. This will be extremely promising if these experimental studies translate into clinical trials. These results however need to be taken with caution and a direct extrapolation to humans is still risky. Indeed, cure rates observed in the last four Chagas disease clinical trials do not reach 100% parasitological cure as is the case in animal models. This raises further questions related to non-responders to current treatment and the reason for these observations.

A number of studies have attempted to establish a link between geographical location, *T*. *cruzi* strain and type of pathology elicited with variable observations [37], [38]. A recent systematic review however showed no clear evidence for such an association as well as no link between *T*. *cruzi* genotype and chronic morbidity, risk of reactivation or mode of transmission [39].

The unanswered questions in the CD field (see Box 1) complicate Research and Development (R&D) itself for the disease. The main limitation on CD R&D is defining the types and properties of drugs that are needed given that the disease and its progression are not yet fully understood. In addition, a lack of tools for measuring drug efficacy compounds is a problem for CD R&D.

Although a lot of data has been generated in the Chagas disease field, it is often inconsistent, difficult to compare or has design flaws which undermine the validity of the results. Evidence-based medicine should be derived from solid data obtained from randomized clinical trials, but very often in the CD area, hypotheses are based on single patient observations or small, open, non-randomized clinical studies. A recent Cochrane systematic review focusing on trypanocidal drugs for chronic asymptomatic *T*. *cruzi* infection concluded that despite the evidence that trypanocidal treatment reduced parasite related outcome, the low quality and inconsistency of the data for patient outcomes should be treated with caution [40]. For these reasons, data generated so far in the Chagas field is insufficient to enable us to provide clear answers to the fundamental questions raised.

Consequently, it is difficult to define the Target Product Profile (TPP) or the desired properties (Target Candidate Profile or TCP) of future drugs for the disease. Depending on the Target Population (e.g. asymptomatic patients in the indeterminate stage of the disease or patients in the chronic stage with target organ involvement) the required drug properties will likely be different. There is a real need to conduct a thorough risk/benefit analysis of treatments for these two categories of patients as treating asymptomatic patients, only a small proportion of which will go on to develop chronic disease, with a drug which shows significant side effects will limit coverage and reduce impact. Depending on the patient category, clinical benefit will have different meaning.

The major goal of treatment for Chagas disease patients in the indeterminate stage is to halt progression to the clinically determined form, in particular avoiding the manifestation of cardiac disease — the most frequently observed clinical outcome — or megaesophagus/megacolon.

Disregarding the debate surrounding the different hypotheses related to Chagas disease progression, an important step for the treatment of an infected patient is to eliminate the parasite from the body. There is some evidence — although nothing definitive — that eliminating the parasite, the etiological factor, is important [34]. Moreover, it has been shown that treatment of infected mothers with anti-parasitic drugs reduces the frequency of congenital transmission making anti-parasitic treatment a very important tool from a public health perspective [41].

These observations suggest that parasitological cure can have an impact on Chagas patients. This concept — along with vaccine development — is currently the basis of all drug discovery programs. So far the development of drugs targeted at eliminating the *T*. *cruzi* parasite is driving the R&D process in the field.

## 3 How do we clinically assess the efficacy of a Chagas drug?

The lack of easy to use and sensitive tests to assess etiologic treatment efficacy has hampered the clinical development for Chagas disease. Typically, serological tests (ELISA, IFA) based on different antigens are used to diagnose chronic patients. Conversion to negative serology is currently the only test available to assess parasitological cure. However, this negative seroconversion can take years to decades after treatment to occur in adult population and is therefore not adequate as an endpoint for clinical trials. There is therefore a need to identify a surrogate marker(s) for the absence or reduction of parasite load which is quicker and more sensitive than seroconversion.

The current strategy for clinical trials is to use PCR to measure parasitaemia as the primary endpoint. This means that inclusion criteria require a positive PCR in addition to serology. A big effort has been made to standardize and validate PCR as the method of choice to detect *T*. *cruzi* parasite in the blood [42]. This is indeed a challenge as parasite load in chronic Chagas patients is very low. Optimization of the method with increasing sampling frequency as well as the development of a quantitative PCR method has led to better results [43]. The use of PCR in combination with another technique (e.g. xenodiagnosis), if practicable, could be a way to improve parasitaemia assessment.

However, PCR does have clear limitations; in Proof of Concept Phase 2 clinical trials (PoC), although very useful, fluctuating parasitaemia means that an individual can periodically be under the limit of detection which raises the issue of sensitivity. Thus PCR only gives an idea of treatment failure and not treatment efficacy. *T*. *cruzi* parasite diversity, the possibility of patients being infected with a mixed population and a lack of data showing that parasitaemia is representative of tissue parasitism are also problematic. An additional issue with PCR is that not all Chagas patients will be PCR positive; indeed, a study showed that depending on the region, between 20% and 60% of Chagas patients are PCR negative [44].

As mentioned earlier, PCR is acceptable for Phase 2 PoC to measure treatment failure, but for Phase 3, it may be a regulatory requirement to be able to accurately measure efficacy of treatment. The current strategy for clinical trials is to balance knowledge and urgent medical need. Do we believe that PCR is good enough for registration? Is it scientifically sound? Can we accept the absence of parasites in the blood as a measure of cure, when studies have shown that this is not necessarily the case? What about those patients that are not PCR positive who might represent the majority of cases?

The question then arises: what else if not PCR? So far, attempts to look at other potential markers of treatment efficacy such as anti-*T*. *cruzi* antibodies (lytic antibodies, anti-Tc24, multiplex serological analysis to name but a few), or the host response (Anti-*T*. *cruzi* T-cell responses, transcriptomics, differential gene expression and non-specific markers) have not led to a major breakthrough [45], [46].

One area that has been explored and showed promise is the identification of potential markers of cure using proteomic platforms. These analyses compared the proteomic profiles and signatures from the sera of infected patients with uninfected individuals and infected patients undergoing treatment in order to identify selective markers for assessment of cure. A recent study has identified Apo-lipoprotein A1 and fibronectin fragments as markers with potential and these are currently being further assessed as the first biomarkers predictive of cure in CD patients [47].

Prospective studies with longer follow-ups are needed for the appraisal of biomarkers assessing clinical cure after therapy. Most probably the use of more than one biomarker will be needed to assess the efficacy of a given treatment.

Another area of potential interest is related to the identification of markers of disease progression and prognostic factors. If one could predict which *T*. *cruzi* infected people will develop the disease, then it would be possible to treat only those at risk. Preliminary attempts have been made trying to identify candidate genes associated with the progression of the disease. Results from a Genome Wide Association Study (GWAS) using the well-established REDS-II cohort of Chagas patients suggested that both cardiovascular- and immune-related polymorphism in some genes of interest could be associated with the genetic predisposition to chronic Chagas cardiomyopathy [48]. Another study found an association between HLA haplotype and resistance to chronic Chagas disease [49]. In a retrospective cohort study of cardiomyopathy incidence among previously asymptomatic Chagas patients, factors such as male sex, history of ECG abnormalities, among others, were associated with prognostic markers for cardiomyopathy [50]. Clearly this is an area of research that merits further investigation.

## 4 Chagas disease drug discovery and development: a very dynamic landscape

Notwithstanding all the challenges described above, there is no doubt that the Chagas R&D environment has been very active in the last 5 years. The recent clinical trials — CHAGASAZOL (NCT01162967), STOPCHAGAS (NCT01377480), E1224 (NCT0148228), BENEFIT (NCT0123916) — the first in 40 years, have contributed important new data and played a major role in highlighting some key issues in the field [51], [52], [53], [54]. It has been a turning point in the field that has changed our thinking and approach to Chagas disease. These trials confirmed the potential of benznidazole as a drug to induce parasitological cure in indeterminate Chagas patients (or lack of treatment failure to be more precise). They also demonstrated the failure of azoles (repurposed fungal CYP51 inhibitors) to induce parasitological cure as assessed by PCR after one-year follow-up. However, and very often comparing with the antibiotic field, controversy still persists whether sterile cure is needed (i.e. parasite load reduction would be enough), to prevent progression of the disease. The length of follow-up of Chagas clinical trials to date is too short to make any conclusion on that question. In the absence of a clear-cut answer, some efforts aimed at developing *T*. *cruzi* specific CYP51 inhibitors are still being considered [55], [56]. These studies have also highlighted a lack of sound interpretation of scientific rationale, shown how difficult it is to break dogma and the importance of an integrated research pipeline to speed up the delivery of future Chagas disease treatment candidates. This in turn has led to a change in the drug discovery approach and screening cascade that has been described elsewhere [57]. The “back-translation” of this clinical research data led to the development of murine models of Chagas disease which are able to predict the clinical outcome of posaconazole and benznidazole treatment [58], [59]. This has been a much needed new tool as the lack of appropriate predictive animal models and the absence of standardization of these models has further hampered the progression of potential new drugs for CD [60], [61], [62], [63]. More results coming from future clinical trials with either new drugs or different regimens of current standards of care (benznidazole and nifurtimox) will be necessary to further support or invalidate the currently favored Chagas murine models. Efforts are being made to, on the one hand, standardize assays, models and screening cascades across the different investigators in the field, while on the other hand identifying and validating novel targets using new tools such as Bioluminescence Imaging (BLI), whole genome sequencing, and -omics. Knowing the target of a potential drug candidate issued from phenotypic screening could allow for better study design in vivo with a particular treatment regimen but also improved access to new chemical entities through target-based screens [64], [65]. Altogether, these new developments should lead to new potential candidates to move forward into clinical trials.

## 5 Priorities/needs: next steps

We should strive to do the “right” experiment and design the best studies to be able to answer the key questions which are needed to develop the best drugs possible for Chagas patients. Given the amount unknown in the field this is not an easy task. Box 2 depicts a few priorities that might be worth considering for the future.

The immediate priority should be to better characterize the two current drugs available for the disease with a specific aim to decrease side-effects and allow for better compliance. Clinical trials looking at reduced treatment length and doses of benznidazole are due to start shortly. In addition, results from recent studies will validate or invalidate the current murine disease models.

A global and concerted effort is needed to identify surrogate markers of treatment efficacy and possibly also predictors of disease progression. This effort is key for the development of new drugs despite the many associated challenges such as the need for extended patient follow-up or access to well preserved sera samples in trials. This is worthwhile for practitioners too, for whom being able to propose rapid and appropriate information to the patient on the treatment outcome with the ultimate aim of improving compliance, is a priority.

Clearly, a switch from the currently fragmented basic and clinical research landscape to a broader collaborative approach with clear focus is needed. This is required in order to generate solid data answering the key Chagas questions which would enable the design of the best drug for Chagas patients as well as the assessment of their efficacy.

## 6 Summary and outlook

There is still a significant gap in the Chagas disease R&D landscape and numerous hurdles to overcome. We are dealing with a complex parasite and complex disease with a lot still unknown. Not enough clinical research is feeding back into drug discovery and the lack of markers of cure or treatment efficacy is a major limitation to current R&D efforts. This all adds to the challenge of developing drugs, a process which is already very complex resulting in an extremely high attrition rate for Chagas drug discovery.

Although major changes have shaken the Chagas R&D landscape during the last 5 years and there is more confidence today, a lot remains to be achieved. There is a need to redefine R&D priorities for Chagas disease and act accordingly; in particular, taking care in the design of studies in order to make sure that solid data is generated, giving an answer to key questions relevant for a better understanding of the disease and the potential outcome for the patient. We have to challenge current thinking, define the next goals and priorities for Chagas disease as well as the strategy to attain these goals.

All this needs not only a broader collaborative approach but also a concerted effort given the limited available resources and the range of questions to answer. In short, this means more basic and clinical research to solve the puzzle piece by piece which will require the Chagas research community as a whole pulling together with a spirit of collaboration. Finally, Chagas disease is also a political challenge that will need to be addressed; serious measures and processes are required to overcome the current situation and barriers to access to treatment as currently too few patients are being treated [17], [66], [67]. This is a sine qua non condition to ensure that CD patients will benefit from new R&D developments for that disease.

## Box 1 Unanswered questions about Chagas disease.

• Is killing *T*. *cruzi*, the etiological factor, enough? Is there any proof that clearance of parasites will prevent development/progression of the disease?
• What is the role of innate and acquired immune responses for parasite persistence? What is our understanding of the role of host/parasite interactions in the pathogenesis of CD?
• Does duration of infection before treatment have an impact on the treatment outcome?
• Where does *T*. *cruzi* persist in chronically infected hosts?
• Why will some infected people develop the disease (up to 30–40%) and others not? Is progression of the disease due to an accumulation of damage? Is it time-dependent? Are there genetic factors which play a role?
• Is there a relationship between *T*. *cruzi* strains and their tropism with the type of pathology developed (megacolon and cardiomyopathy) and geographical location? What factors determine the development of either or both pathologies?
• How do we define cure in patients in the indeterminate stage of the chronic phase of the disease? How is clinical benefit defined for this category of patients and how do we measure it?
• Which patients fail to be parasitologically cured following treatment with the current drugs?

Alt-text: Box 1

## Box 2 What should be the priorities for the next steps in Chagas disease R&D.

• Determine the optimal regimen of the current drugs — benznidazole and Nifurtimox — in chronic indeterminate patients
• Follow-up cohort of indeterminate patients to assess/understand the impact of treatment on progression of the disease
• Need for surrogate markers of parasitological cure/treatment efficacy possibility markers predictive of disease progression
• More new drugs/chemical classes in clinical trials PoC; fill the pipeline with drug candidates with new Mechanisms of Action (currently there are all nitroheterocyclic compounds bio-activated through *T*. *cruzi* Nitroreductase I)
• Need for more research and access to new chemical entities

Alt-text: Box 2
